# Peptide-specific chemical language model successfully predicts membrane diffusion of cyclic peptides

**DOI:** 10.1101/2024.08.09.607221

**Published:** 2024-08-09

**Authors:** Aaron L. Feller, Claus O. Wilke

**Affiliations:** † Interdisciplinary Life Sciences, The University of Texas, Austin; ‡ Department of Integrative Biology, The University of Texas, Austin

## Abstract

Biological language modeling has significantly advanced the prediction of membrane penetration for small molecule drugs and natural peptides. However, accurately predicting membrane diffusion for peptides with pharmacologically relevant modifications remains a substantial challenge. Here, we introduce PeptideCLM, a peptide-focused chemical language model capable of encoding peptides with chemical modifications, unnatural or non-canonical amino acids, and cyclizations. We assess this model by predicting membrane diffusion of cyclic peptides, demonstrating greater predictive power than existing chemical language models. Our model is versatile, able to be extended beyond membrane diffusion predictions to other target values. Its advantages include the ability to model macromolecules using chemical string notation, a largely unexplored domain, and a simple, flexible architecture that allows for adaptation to any peptide or other macromolecule dataset.

## 1 Introduction

Therapeutic peptides have gained attention as promising clinical agents due to their diverse chemical properties and their efficacy in treating a variety of diseases.^1–3^ Advances in peptide production and modification technologies have facilitated the development of drug targeting strategies involving cyclization reactions and the alteration of both side-chain and backbone chemistries beyond those found in nature.^4–11^ Existing deep-learning models have been successful in modeling small molecule drugs and large proteins.^12,13^ Additionally, protein language models have been fine-tuned to for inference tasks on natural peptides.^14^ However, these frameworks fall short in peptide drug discovery applications due to limited diversity in pretraining data and restrictions imposed by natural amino acid vocabularies. The increasing diversity in synthetic peptide chemistries necessitates robust computational methodologies capable of accurately encoding and predicting their properties.

The development of peptide drugs for intracellular targets has been a goal of the medical community for some time.^15,16^ While models for predicting membrane penetration in small molecule drugs and natural, linear peptides have achieved high accuracy,^7,17,18^ they struggle with cyclic or modified peptides. The predictive modeling of these complex molecules has often relied on molecular dynamics simulations, which are computationally expensive. ^19^ Moreover, the membrane permeability of cyclic peptides does not conform to simple rules like Lipinski’s rule of five for small molecules.^20,21^ Therefore, a comprehensive model for peptide-based drug discovery must encode peptide backbone modifications, various natural and unnatural amino acids, and diverse side-chain modifications and cyclizations. One approach to address this problem is to apply chemical string notation, such as the Simplified Molecular-Input Line-Entry System (SMILES), to describe diverse and pharmacologically relevant peptides.

Here, we present PeptideCLM, a peptide-specific chemical language model capable of encoding peptides with chemical modifications, non-canonical amino acids, and cyclic structures. This model uses masked language modeling techniques to train on a curated database peptides and small molecules. Our model achieves higher predictive accuracy for membrane diffusion than existing chemical language models. By employing chemical string notation, PeptideCLM opens a domain of modified macromolecule modeling using language modeling for biological deep learning. Additionally, the model’s architecture is designed for ease of implementation and interpretation, making it a versatile tool for peptide research and drug discovery.

## 2 Methods

### Pretraining dataset generation.

**Figure 1:**
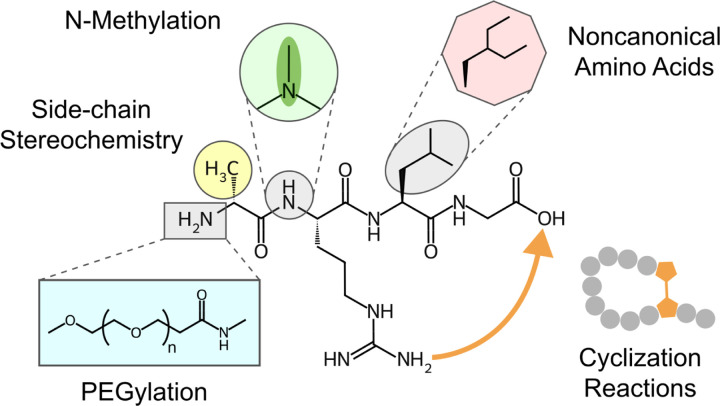
Example modifications of the tetrapeptide Ala-Arg-Leu-Gly. The peptide structure is depicted with five modifications used for synthetic data generation: (clockwise) N-methylation of a backbone amine, substitution of alanine for the synthetic amino acid diethylalanine, cyclization of asparagine to the carboxyl terminus, PEGylation of an amino group, and altered side-chain sterochemistry.

In this study, two distinct datasets were curated for chemical language model (CLM) pretraining. The first consists of 10 million small molecules from PubChem,^22^ as released with the ChemBERTa model,^23^ and 2.2 million small molecules from SureChEMBL,^24^ filtered for single-molecule entries. The second dataset consists of 825,632 peptides from SmProt,^25^ a database of small proteins (<100 amino acids) annotated from ribosome profiling datasets on 8 species, and 10 million modified peptides (Figure 1) generated with a modified version of CycloPs,^26^ a cyclic peptide generator. We updated CycloPs to Python 3 and incorporated 100 unnatural amino acids from SwissSidechain.^27^ All amino acids were included with both levo and dextro chiral conformations, encoded into peptides using single character notation with uppercase characters representing the L-conformation and lowercase representing the D-conformation. Cyclization of the peptide chain was done in a rule-based manner, where all side chains that are able to perform cyclizations were included as options.

The generated peptides consist of 90% canonical amino acids and 10% unnatural amino acids, 10% dextro-chiral alpha carbons, N-methylation on 20% of backbone amines, and PEGylation with 1–4 monomer lengths at random positions on 10% of the peptides. N-methylation was chosen for its ability to increase membrane permeability,^28^ while PEGylation reduces renal clearance, resulting in a longer half-life.^29^ Cyclization reactions were attempted for all generated peptides, randomly selecting from head-to-tail, sidechain-to-sidechain, sidechain-to-head, sidechain-to-tail, disulfide, or uncyclized (linear) configurations. If cyclization was not possible given the available amino acid residues, the molecule was left linear. All molecules were represented as strings using Simplified Molecular-Input Line-Entry System (SMILES).^30^

### Tokenization scheme.

**Figure 2:**
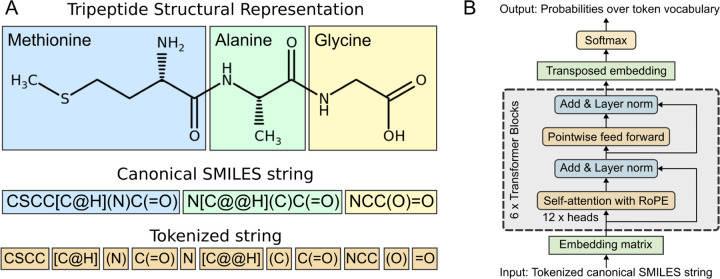
Schema for tokenization of a tripeptide and implementation of a BERT-style architecture for encoding peptides. (A) 2D-structure representation colored by amino acid. SMILES canonical representation generated using RDKit with same coloring. Representative tokens for input into a PeptideCLM. (B) The achitecture for the RoFormer BERT model used as the framework for PeptideCLM. The model contains six transformer blocks with twelve attention heads per block, post-layer normalization, and a standard language modeling head with a softmax distribution over the vocabulary index for token prediction.

A custom tokenization method, derived from SMILES Pair Encoding,^31^ was developed to optimally represent peptide chemistries from SMILES strings (Figure 2A). A pretokenizer first identified multi-character and single-atom tokens, including Br and Cl as distinct from B and C. Subsequently, token assignment of chemical motifs was performed through n-gram analysis of up to 5 characters over the 10 million PubChem SMILES strings. This process identified a total of 581 unique tokens, resulting in 586 tokens when including the 5 special tokens [PAD], [UNK], [CLS], [SEP], and [MASK]. Among the 581 tokens, 53 are single-character tokens, 150 are multi-character tokens (e.g., CCC, Clc, occ), 124 are bracketed ions (e.g., [Ag^+^], [Ag], [Al^−3^]), and 254 are parenthetical motifs (e.g., (/CCl), (=C), (CCCN)).

### Model architecture and pretraining.

PeptideCLM is a BERT-style transformer architecture with Rotary Position Embedding (RoPE) based on the RoFormer implementation described by Su (2024).^32^ Our implementation of RoFormer employs 12 attention heads and 6 layers (Figure 2B), with a hidden embedding dimension of 768 and an intermediate feed-forward layer size of 3072, matching the configurations used in GPT-3^33^ and BERT.^34^

With this architecture, three models were trained to assess the impact of pretraining data. A model with the full pretraining data was trained for 10 epochs on the combined dataset of small molecules and peptides. Two additional models were trained solely on the peptides or small molecule portion of the pretraining data. These two models were trained for 20 epochs to reach a similar number of training steps to the full dataset. Training was conducted using a masked prediction task, wherein 15% of the sites were masked. Each sequence underwent an 80/10/10 split for masking, corrupting with a random token, or leaving the sequence unmasked, respectively, as described in the BERT methodology.^34^ The hyperparameters for pretraining PeptideCLM were batch size of 64 and a learning rate of 5 × 10^−5^, chosen based on preliminary experiments indicating it provided a good balance between convergence speed and stability. The holdout dataset for validation comprised 0.5% of the full model and 1% of the chemical and peptide models, amounting to roughly 115k SMILES strings per model. Validation was performed at intervals corresponding to every 20% of an epoch to ensure consistent model performance and early detection of potential overfitting. The final checkpoint was used for downstream application.

### Finetuning chemical language models.

First, CycPeptMPDB was reduced to only the results from PAMPA, as the database also includes results from Caco2, MDCK, and RRCK. All data points that were categorized as ‘undetectable’ and marked as −10 in CycPeptMPDB were removed due to the potential of aggregation or some other collection error, leading to PAMPA results differing from the true permeability of the peptide.

**Figure 3:**
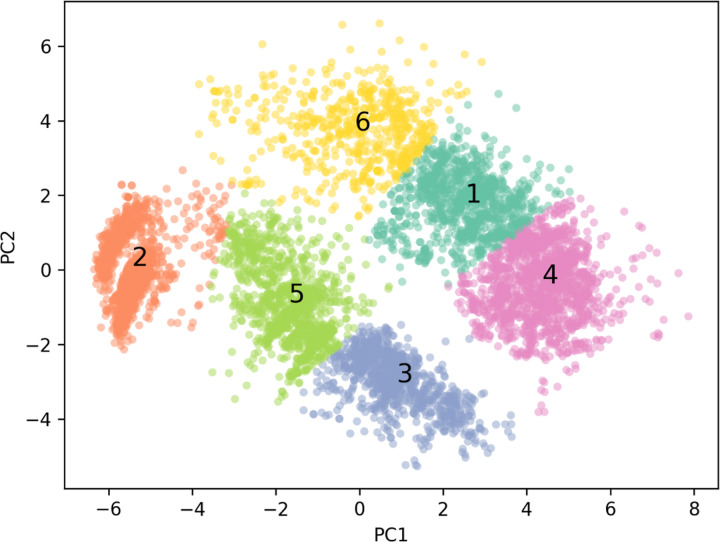
Clustering on principle components of CycPeptMPDB peptide embeddings. Principal Component Analysis (PCA) was performed on peptide embeddings obtained from the pretrained PeptideCLM model. The embeddings were then analyzed using k-means clustering, resulting in six distinct clusters. For model training and evaluation, clusters 1–5 were utilized in a 5-fold cross-validation setup, while cluster 6 was held out and used exclusively for validation purposes.

To replicate a true experimental setting where the molecules examined fall outside the training set, we split the data into 6 groups using k-means clustering on PCA of the embeddings from the pretrained model (Figure 3). The smallest group (*n* = 588) was designated as the test set. For the remaining data, 5-fold cross-validation (CV) was performed, with each group adjusted to contain 949 samples, matching the size of the smallest group in the 5-fold CV setup.

For each architecture, we removed the language modeling head and replaced it with a fully connected feed forward layer with width matching that of the hidden state of the model. Five models were trained using k-fold analysis, with the model weights selected from the checkpoint exhibiting the lowest mean squared error (MSE). The predictions were made on the test set using each model and with mean pooling of predictions from the five models. Training was repeated for five iterations, and the mean and standard deviation were calculated for each architecture and pretraining dataset.

The ChemBERTa models were selected as competitors to PeptideCLM as they are the only publicly available chemical language models with large enough input context to encode the peptides examined in the dataset. Finetuning of all models on our subset of the CycPeptMPDB data was performed with the following hyperparameters: a learning rate of 5 × 10^−6^, a dropout rate of 0.15, and a weight decay of 0.001. The batch size was set to 16, and training was conducted for a maximum of 10,000 steps. Validation was performed at the end of each epoch to monitor performance and assess overfitting.

### Framework and hardware.

All model training and inference were conducted on a system running Linux 20.04.6 LTS equipped with 8x AMD Navi 10 (Radeon RX 5700 XT) GPUs. Pretraining was conducted with all 8 GPUs with fully distributed data parallel. Finetuning was conducted in parallel with multiple models/folds running on a single GPU. The machine learning framework employed for these tasks was PyTorch,^35^ utilizing PyTorch Lightning^36^ for managing the training process. Model architecture and weights have been made available on Huggingface. ^37^

## 3 Results

### Model Design.

With the aim of creating a method for encoding noncanonical peptides, we developed a novel tokenization scheme and pretrained a BERT transformer architecture on a database of small molecules and peptides. By applying self-attention across all input tokens, we generated a fully-connected graph with connections learned by the network during pretraining. The resulting model can embed peptides with various modifications and non-canonical amino acids by representing macromolecules as SMILES strings.

In comparison to the architecture used by ChemBERTa, we increased the context length of the model from 512 to 768 to allow our model to encode peptides and small proteins of roughly 100 amino acids. We replaced static sinusoidal positional embeddings with rotational embeddings on the query and key matrices as developed in RoFormer, ^32^ which has been shown to reduce perplexity for protein language modeling in ESM-2.^13^ The vocabulary for tokenization was defined by analyzing n-grams of up to 5 characters to identify common parenthetical insertions and bracketed ions, a method similar to byte-pair encoding^38^ used in natural language modeling. We included the top n-grams of up to three characters without parenthesis or brackets. This process generated 581 unique tokens, providing a higher compression rate for peptides compared to single character tokenization used in the ChemBERTa models^23,39^ models. Notably, standard byte-pair-encoding did not improve pretraining for ChemBERTa-2.^39^

### Impact of synthetic data for pretraining.

In comparison to the large databases of protein sequences and small molecules, there is significantly less peptide data available for pretraining. The use of synthetic data has been successful for CLM training as in the use of the generated small molecule database ZINC22^40^ for pretraining the BERT-style model MoLFormer.^12^ To supplement the limited amount of available peptide data, we generated 10 million randomized peptide sequences with a modified CycloPs^26^ to supplement our pretraining dataset.

With this implementation, 10 million SMILES sequences were generated for peptides of length 10–100 amino acids with 90% canonical amino acids, 10% noncanonical amino acids, and 10% overall D-conformations for both canonical and noncanonical. This number was chosen to create a balance between the two datasets of small molecules and peptides and to increase the number of tokens to near 100x tokens to parameters (4.6 billion tokens) with the goal of improving pretraining loss.^41^ The sequences included an equal number of possible cyclization reactions: linear (non-cyclized), head-to-tail, sidechain-to-sidechain, head-to-sidechain, sidechain-to-tail, and disulfide-bridge. Following generation of the SMILES database, sequences were modified to include a single PEGylation of a 1–4 PEG monomer chain in 20% of the sequences and addition of N-methylation to 20% of sites in 20% of the sequences.

**Figure 4:**
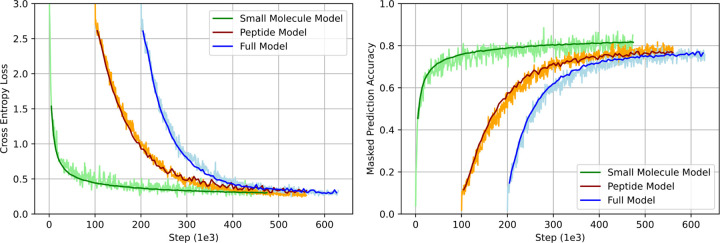
Cross-Entropy Loss and Macro-Averaged Accuracy in Training a BERT-Style Model with Masked Prediction. Cross-entropy loss (left) is computed between the softmax of the token probabilities and the one-hot encoded original sequence. The loss is determined as the sum of the logarithmic loss −(*y* log(*p*) + (1 − *y*) log(1 − *p*)) across all masked positions. Macro-averaged accuracy (right) is calculated as the average accuracy, averaged across classes. The validation set comprised a randomly selected 0.5% of the full dataset, or 1.0% of the peptide or small molecule datasets. Peptide and Full models are shifted right 100^3^ and 200^3^ steps, respectively.

Three models were pretrained to evaluate the impact of the synthetic peptide data on pretraining and the downstream task of predicting membrane diffusion. Results from the pretraining task showed that training the model with small molecules from Pubchem and SureChEMBL led to higher maximum accuracy and lower cross-entropy loss (CEL) (Figure 4). The peptide-only model achieved the poorest accuracy and loss. Pretraining on a combined dataset of peptides and small molecules resulted in CEL values that were intermediate between the two individual models, achieving a final accuracy comparable to the peptide-only model. This result is expected, as randomization of peptides results in some tokens that are impossible to correctly predict. For example, the chirality of the *α*-carbon cannot be learned, as the chirality was selected randomly and cannot be inferred from the rest of the sequence.

### Finetuning on membrane diffusion, as measured by PAMPA.

**Figure 5:**
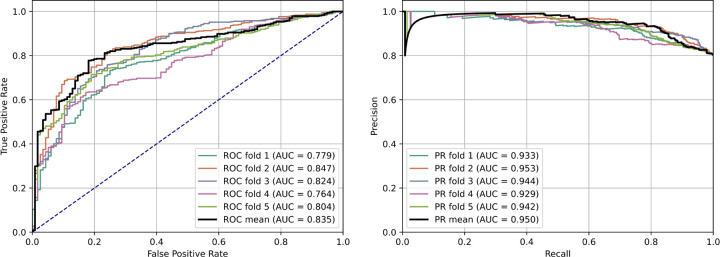
Receiver-operator curves and precision-recall curves of finetuned PeptideCLM for predicting membrane penetration. Receiver-operator curves (Left) and precision-recall curves (right) of the finetuned PeptideCLM model using 5-fold cross-validation. The mean curve is generated by taking the mean pooled prediction of all five models.

The performance of PeptideCLM, ChemBERTa-2, and ChemBERTa were evaluated on their ability to predict the membrane diffusion of cyclic peptides using k-means cluster split from PCA of pretrained PeptideCLM embeddings. Model evaluation was done by finetuning each model with an added fully connected layer attached to the CLS token for a maximum of 10,000 steps with 5-fold cross-validation. The models were finetuned to predict the logarithm of the diffusion constant in a regression manner. The data was subset into six clusters using k-means, with the smallest group as a holdout for testing. This was performed across 5-folds of the data for up to 20,000 steps, with checkpointing at the end of each epoch. The final checkpoint was selected based on the lowest MSE observed on the validation set. The models were then used to classify membrane penetration by setting a cutoff for *LogP*_*exp*_ of −6.00 (1.0 × 10^−6^ cm/s) or higher for membrane penetrating peptides, and lower for non-penetrating. Final test metrics were calculated as the mean pooled prediction for the five models. This process was repeated five times for each model and the mean and standard deviation across five iterations is reported. Receiver Operating Characteristic (ROC) and precision-recall (PR) curves were generated and area under the curve (AUC) was used for determining success (Figure 5). PeptideCLM was the most accurate model with a ROC-AUC of 0.820 and a PR-AUC of 0.948; finetuning ChemBERTa-2 MTR 77M was the next best model with ROC-AUC of 0.770 and PR-AUC of 0.934 (Table 1).

## 4 Discussion

In this study, we have developed a peptide-specific chemical language model, PeptideCLM, capable of encoding peptides with various chemical modifications, including non-canonical amino acids and cyclic structures. Our model has demonstrated superior performance in predicting membrane diffusion from SMILES strings of an embedding-based holdout set of cyclic peptides, outperforming the existing chemical language models capable of performing this task, namely ChemBERTa and ChemBERTa-2. The results highlight the potential of this modeling approach in addressing the non-trivial challenges in peptide modeling. In particular, we have highlighted the promise of encoding complex macromolecules using a chemical string notation.

Our results indicate that a chemical language model (CLM), when pretrained on a diverse dataset of natural and synthetic peptides, exhibits an enhanced ability to generalize to out-of-distribution peptides. The high ROC-AUC and PR-AUC scores demonstrate the model’s robustness in predicting membrane permeability, which is crucial for intracellular peptide drug targets. This performance underscores the importance of using a diverse pretraining dataset and a tailored tokenization scheme to capture the intricate details of peptide structures.

The k-means clustering approach used for cross-validation ensured that the model was tested on a representative subset of the data, mimicking real-world scenarios where peptides may vary significantly in structure. The consistent performance across different clusters demonstrates the model’s ability to handle diverse peptide modifications and suggests its applicability to a wide range of peptide-based applications.

Previous research on language models in biology has primarily focused on small molecules^23,39^ or large proteins,^12,42^ thereby leaving a gap in the modeling of mid-sized peptides. Protein language models have been adapted for use with peptides, ^14^ but cannot encode modifications beyond the 20 natural amino acids or cyclizations. Our approach bridges this gap by leveraging the flexibility of SMILES notation and the powerful representation learning capabilities of transformer models.

While the ChemBERTa models are effective for generating representations of small molecules, our results indicate reduced performance when modeling peptides. ChemBERTa is a chemical language model trained via the self-supervised task of masked language modeling (MLM). Our architecture is very similar to their work, with some changes that include a customized peptide token vocabulary, rotational embeddings on the query and key matrices, and an expanded pretraining dataset that incorporates synthetic data for 10 million peptides with cyclizations and chemical modifications. In comparison, ChemBERTa-2 has a reduced parameter count yet outperforms ChemBERTa. This advance seems to arise from an alternative pretraining task of multi-task regression on computationally derived molecular descriptors. Notably, ChemBERTa-2 uses linear attention, rather than the dot-product attention, reducing the computational complexity at the cost of accuracy.

A structural deep learning approach^43^ was conducted on CycPeptMPDB^44^ that used a hybrid model architecture, including various chemical, 2D, and 3D descriptors. This model achieved similar results to our model when predicting on a random split of the data. We could not make a comparison on our embedding-based data split because the model was not been released at the time of this study. The structural modeling approach used pose estimation, requiring peptide structure predictions. Another recent work explored cyclic peptide conformation changes when crossing a membrane. ^45^ Because chemical language models are structure agnostic, with attention being learned during training, they may provide an advantage by creating internal representations that mimic pose estimation from use of a fully connected graph, as in PeptideCLM. A systematic analysis predicting membrane penetration for various assays was also reported using standard machine learning methods such as random forest and gradient boosting methods.^46^ This study resulted in an R^2^ of 0.658 for PAMPA in CycPeptMPDB.^44^

Due to computational constraints, we were limited to training a model with a small number of parameters in comparison to state-of-the-art protein language models. For sequence-only models, ESM-2^13^ showed a decrease in pretraining loss for model sizes up to 15 billion parameters. A higher complexity model, ESM-3^47^ showed improvements in model size up to 1 trillion parameters. With additional synthetic data generation, it is imaginable that an increase in model parameters would produce a model that achieves higher accuracy.

Due to using standard self-attention, we limited our context window to keep the memory during training manageable for both our pretraining and for an end-user. It may be worth exploring if a larger context window combined with linear attention might be useful. Another way to resolve the issue of memory consumption would be to use a state-space model to generate embeddings.

The pretraining dataset, while large and diverse, may still not capture the full spectrum of possible peptide modifications found in nature and synthetic biology. Additionally, the current model architecture, although effective, could benefit from further optimization, such as incorporating pre-layer normalization to enhance training stability. The application of linear attention to a model pretrained on peptides is worth investigating to see if in improves training loss.

A further limitation is the reliance on SMILES strings for peptide representation. While SMILES provides detailed descriptions of chemical structures, it can be cumbersome for representing large and highly complex peptides. Future work could explore alternative representations, such as graph transformer-based approaches, to capture the spatial and functional aspects of peptide structures more effectively. Additionally, datasets with cyclic peptides are limited, however, our model could be applied to linear, canonical peptides. We hope to explore this in the future.

## 5 Conclusion

PeptideCLM represents a significant advancement in peptide modeling, offering a flexible and powerful tool for predicting diverse properties of peptides. Our method of tokenization and model design is an alternative modeling approach in the field of peptide-based drug discovery. Practical application of language models to noncanonical peptides could be used as an aid to guide drug discovery. The insights gained from exploring alternative modeling approaches to peptides will guide future research and development, expanding the repertoire of computational methods available for therapeutic peptide design.

## Figures and Tables

**Table 1:** Performance comparison of ChemBERTa and PeptideCLM models.

	Parameters	5-Fold Ensemble	
		ROC-AUC	PR-AUC
**ChemBERTa-1**	44M		
MLM 10M		0.662 ± 0.014	0.880 ± 0.010
**ChemBERTa-2**	3.4M		
MLM 5M		0.701 ± 0.015	0.896 ± 0.017
MLM 10M		0.712 ± 0.014	0.907 ± 0.005
MLM 77M		0.648 ± 0.010	0.878 ± 0.007
MTR 5M		0.724 ± 0.010	0.912 ± 0.006
MTR 10M		0.699 ± 0.012	0.899 ± 0.011
MTR 77M		0.770 ± 0.009	0.934 ± 0.004

**PeptideCLM**	44M		
Randomly Initialized		0.612	0.847
Chemical Pretraining		0.688 ± 0.009	0.899 ± 0.006
Peptide Pretraining		0.736 ± 0.008	0.922 ± 0.003
Full Pretraining		**0.820** ± **0.014**	**0.948** ± **0.004**
