# Supplementary material for: Peptide-aware chemical language model successfully predicts membrane diffusion of cyclic peptides
Source: bioRxiv. 2024 Nov 21:2024.08.09.607221. Originally published 2024 Aug 9. Preprint. [Version 2] doi: 10.1101/2024.08.09.607221 (PMC11326283; doi:10.1101/2024.08.09.607221)
Supplement: Supplement 1 [file media-1.pdf]

Supplemental Information: Peptide-aware chemical language model successfully predicts membrane diffusion of cyclic peptides

1 Supplemental Tables

Table S1: Datasets used in PeptideCLM masked language modeling pretraining. Checkmarks denote whether a dataset was used in training the model.

Data type (curation source)	Count	Full Model	Peptide Model	Small Molecule Model
Generated peptides (CycloPs)	10M	✓	✓	
Natural peptides (SmProt)	825K	✓	✓	
Small molecules (PubChem)	10M	✓		✓
Patented molecules (SureChEMBL)	2.2M	✓		✓
**Total**	**23M**	**23M**	**10.8M**	**12.2M**

Table S2: Dataset details for CycPeptMPDB used in finetuning. SMILES were generated for all peptides and columns represent the average SMILES length, average permeation score, number of peptides considered permeating, number of peptides considered non-permeating, and the total number of peptides for each step of filtering or each cluster.

	Avg. SMILES Length	$\mathrm{LogP_{exp}}$ Avg.	$\mathrm{LogP_{exp}}$ ≥ -5.5	$\mathrm{LogP_{exp}}$ < -5.5	Total
**CycPeptMPDB**	-	-	-	-	7,451
**PAMPA only**	150.7	-5.87	2,854	4,087	6,941
**Without outliers**	151.5	-5.72	3,847	2,854	6,701
**Cluster 1**	125.8	-5.57	767	779	1,546
**Cluster 2**	181.0	-5.90	515	950	1,465
**Cluster 3**	116.2	-6.01	402	1,053	1,455
**Cluster 4**	134.3	-5.80	413	793	1,206
**Cluster 5**	225.3	-5.06	419	116	535
**Cluster 6**	210.0	-5.29	338	156	494

Table S3: Hyperparameters for pretraining and finetuning PeptideCLM.

**Model Architecture**	**Pretraining**	**Finetuning**
Total Parameters	44M	44M
Attention Heads	12	12
Number of Layers	6	6
Hidden Dimension	768	768
Feed-forward Dimension	3,072	3,072
Vocabulary size	586	586
Initializer	Xavier	Xavier
Optimizer	Adam	Adam
$\beta 1$	0.9	0.98
$\beta 2$	0.9	0.98
ϵ	10^{-8}	10^{-8}
Learning rate	5×10^{-5}	5×10^{-6}
Dropout rate	0.1	0.15
Clip norm.	0	0
Layer norm. ϵ	10^{-12}	10^{-12}
Weight decay	0	0.001
Activation	GELU	GELU
Batch size	64	16
Masking	15% (80/10/10)	-
Steps/Epochs	10 epochs (20 for ablation studies)	10,000 steps max.
Precision	16-mixed	16-mixed
Data Strategy	Distributed Data Parallel	Single GPU

2 Supplemental Figures

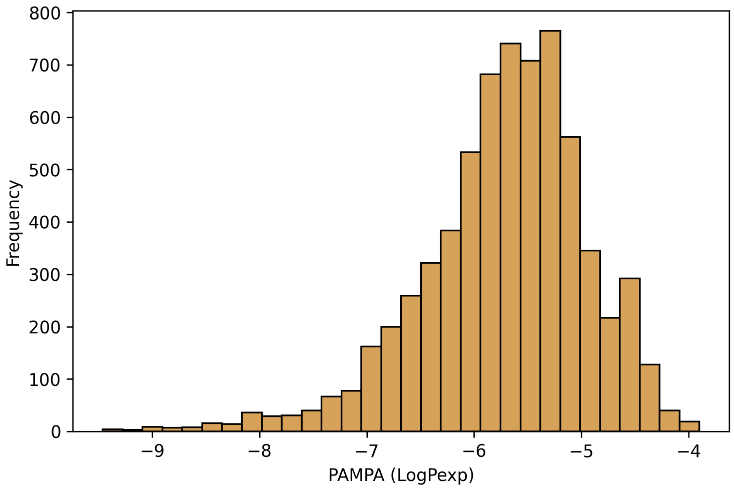

Figure S1: Distribution of LogP_{exp} scores measured by PAMPA assay for cyclic peptides in CycPeptMPDB. Peptides that were undetectable and scored -10 were removed.

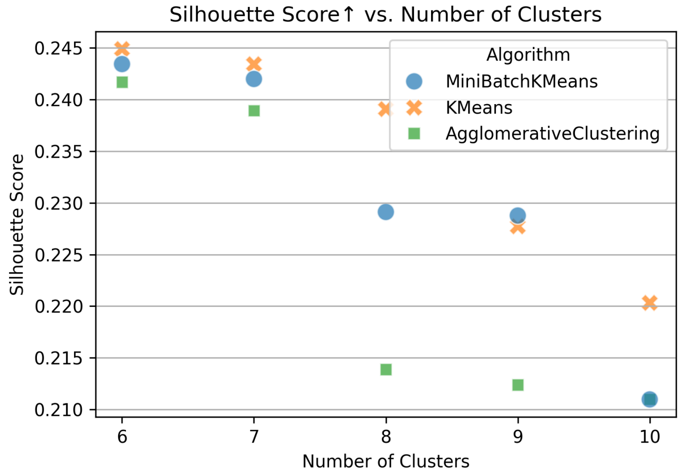

Figure S2: Clustering of PAMPA data was evaluated using Silhouette Score to determine the best clustering method. A higher silhouette score represents higher cohesion and better separation of the clusters. MiniBatchKMeans, KMeans, and Agglomerative Clustering were assessed for sizes of 6 to 10 clusters.

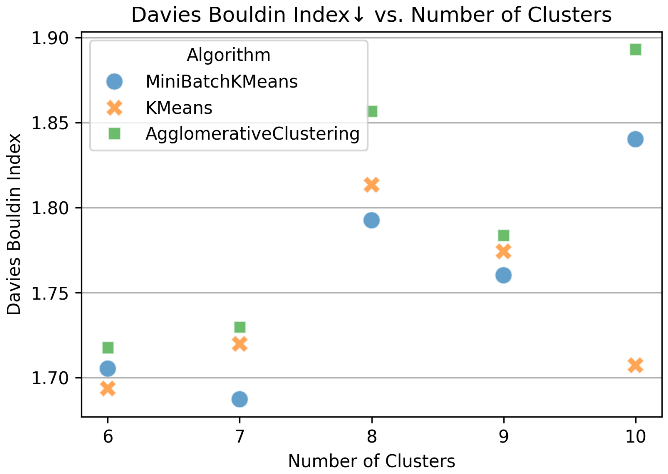

Figure S3: Clustering of PAMPA data was evaluated using Davies-Bouldin Index (DBI) to determine the best clustering method. A lower DBI score shows improved separation and compactness of the clusters. MiniBatchKMeans, KMeans, and Agglomerative Clustering were assessed for sizes of 6 to 10 clusters.

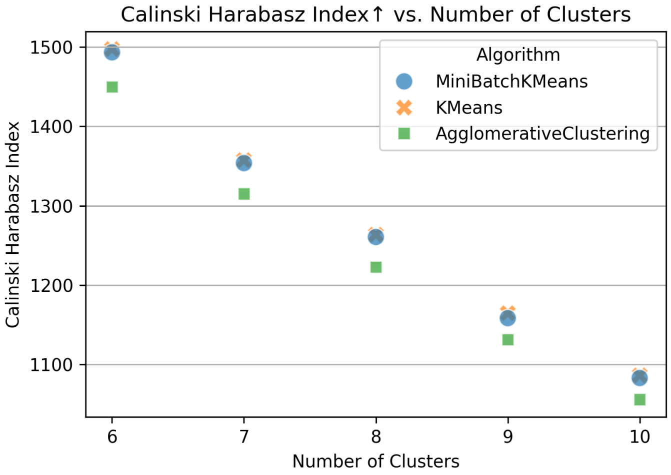

Figure S4: Clustering of PAMPA data was evaluated using Calinski-Harabasz Index (CHI) to determine the best clustering method. A higher CHI score shows improved separation and compactness of the clusters. MiniBatchKMeans, KMeans, and Agglomerative Clustering were assessed for sizes of 6 to 10 clusters.

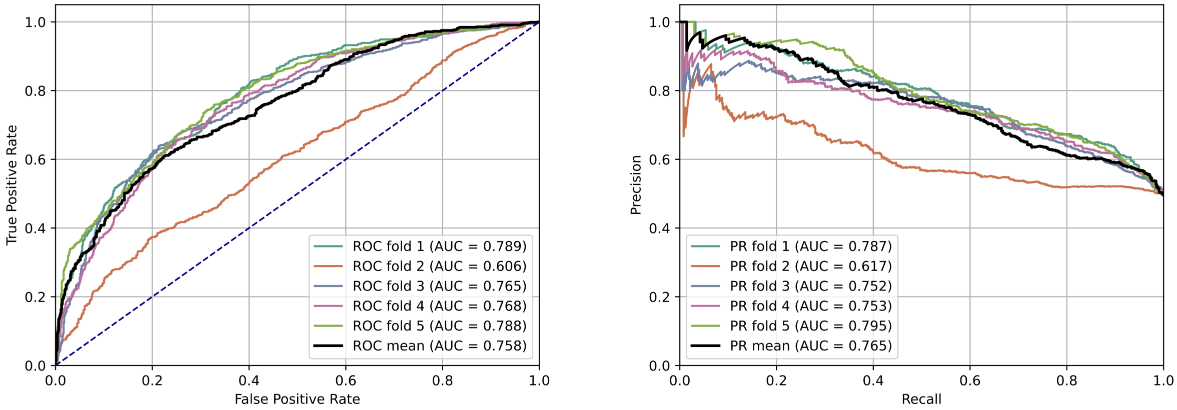

Figure S5: Receiver-operator curves and precision-recall curves for PeptideCLM-23M on holdout test cluster 1.

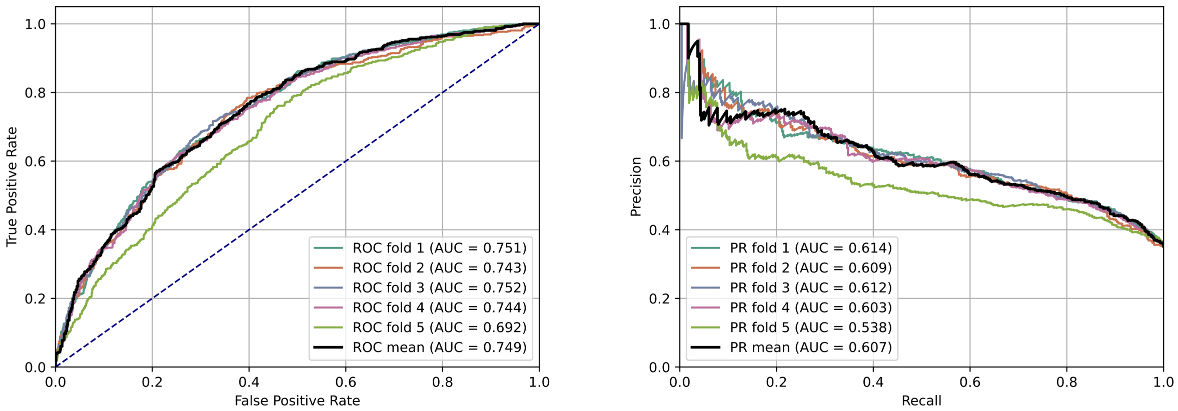

Figure S6: Receiver-operator curves and precision-recall curves for PeptideCLM-23M on holdout test cluster 2.

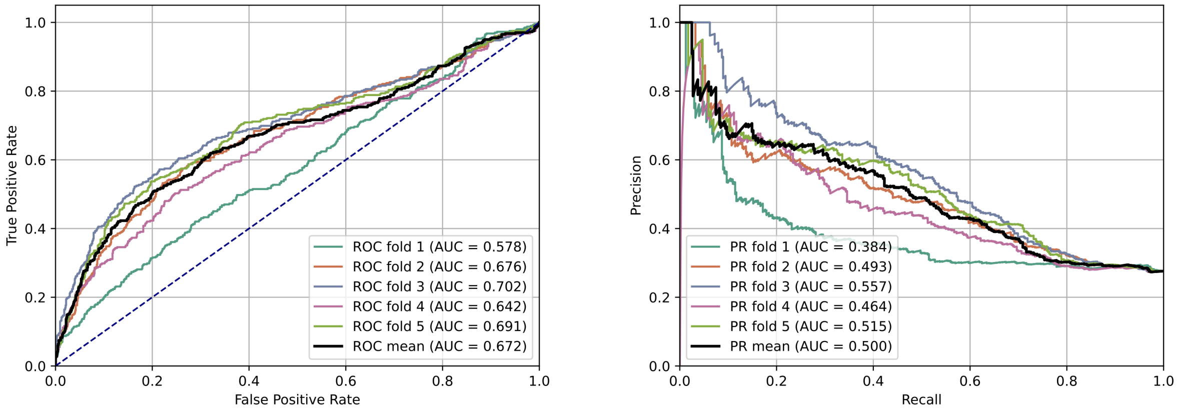

Figure S7: Receiver-operator curves and precision-recall curves for PeptideCLM-23M on holdout test cluster 3.

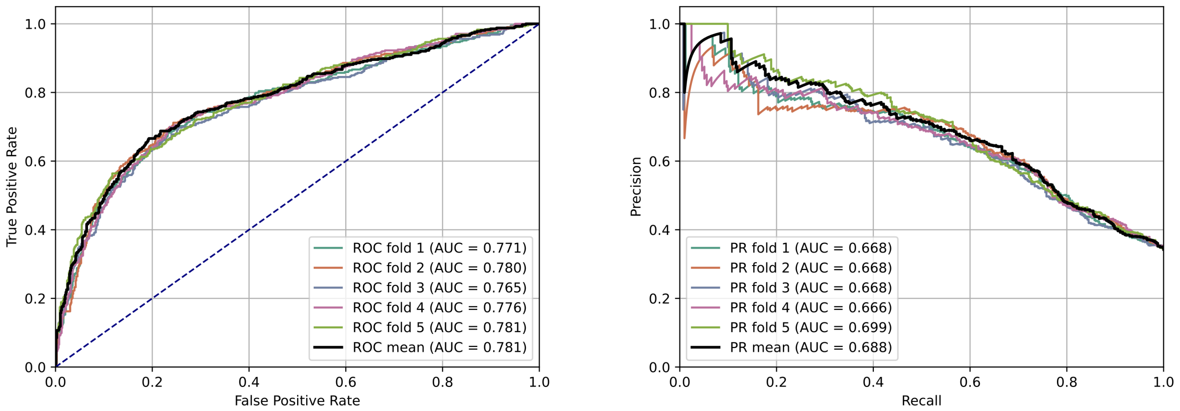

Figure S8: Receiver-operator curves and precision-recall curves for PeptideCLM-23M on holdout test cluster 4.

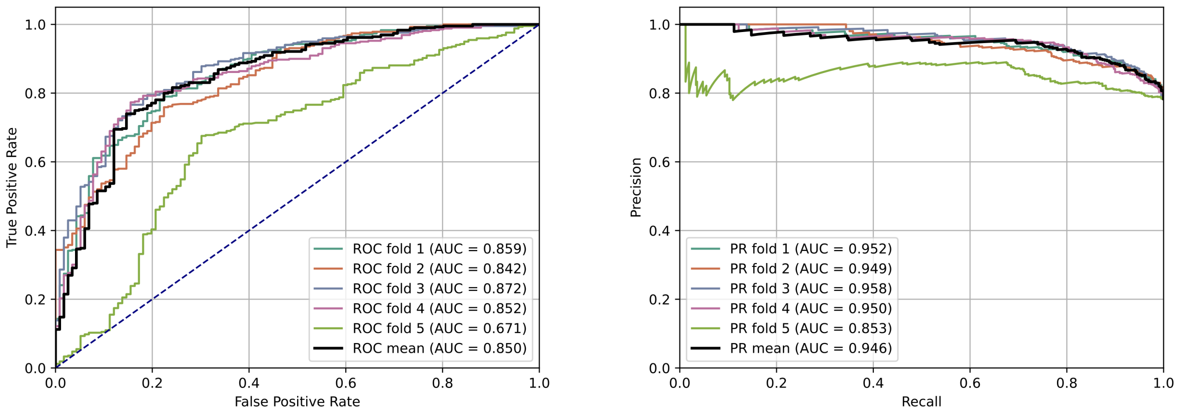

Figure S9: Receiver-operator curves and precision-recall curves for PeptideCLM-23M on holdout test cluster 5.

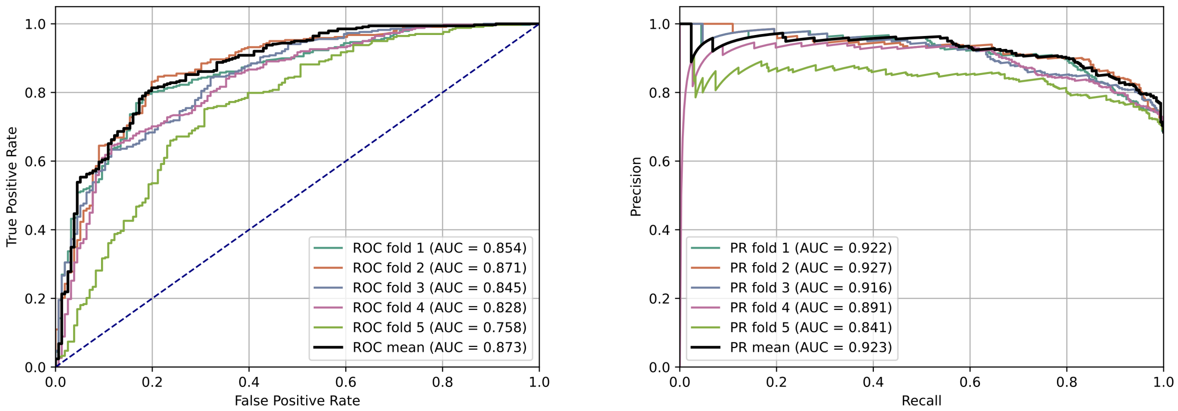

Figure S10: Receiver-operator curves and precision-recall curves for PeptideCLM-23M on holdout test cluster 6.
